# Enhanced resolution of optical genome mapping utilizing telomere-to-telomere reference in genetic disorders

**DOI:** 10.1038/s41431-024-01763-z

**Published:** 2024-12-09

**Authors:** Sofia Banu, Kanakavalli MK, Joel Kiran George, Elizabeth Siby, Rakeshpal Bhagat, Sreelekshmi MS, Siddaramappa J. Patil, Shubha R. Phadke, Divya Tej Sowpati, Karthik Bharadwaj Tallapaka

**Affiliations:** 1 CSIR-Centre for Cellular and Molecular Biology (CSIR-CCMB), Hyderabad, Telangana India; 2 Academy of Scientific and Innovative Research (AcSIR), Ghaziabad, India; 3 Division of Medical Genetics, Mazumdar Shaw Medical Center, Narayana Hrudayalaya Hospitals, Bangalore, India; 4 Department of Medical Genetics, Sanjay Gandhi Postgraduate Institute of Medical Sciences, Lucknow, India

**Keywords:** Laboratory techniques and procedures, Medical genetics, Genetic testing

## Abstract

Reference genomes serve as a baseline criterion for comparison of personal genomes to deduce clinical variants. The widely used reference genome, GRCh38, contains stretches of gaps and unresolved bases particularly in complex regions which could obscure variant discovery. In contrast, the gapless telomere-to-telomere CHM13 (T2T-CHM13) reference genome can be used to assess difficult regions of the genome. Optical genome mapping (OGM), an imaging technique for structural variation identification has improved resolution compared to traditional cytogenetic methods. Our study showcases the utility of the T2T-CHM13 reference genome for enhanced structural variant (SV) detection in complex regions. We illustrate this through two clinical cases, where improved alignment with T2T-CHM13 led to significantly higher confidence scores for critical SVs. We demonstrate improved clinical diagnostic outcomes with the updated T2T-CHM13 reference and advocate its adoption.

## Introduction

Evaluation of whole genomes is rapidly emerging as a standard diagnostic test in rare diseases. While small genetic variants such as SNPs and InDels can be identified by molecular tests and whole exome panels (WES), these technologies are limited in detection of large variants [1]. Large structural variants can have profound consequences in research of Mendelian and complex diseases but are challenging to resolve [2, 3]. Whole genome sequencing using short or long read sequencing and imaging techniques like optical genome mapping (OGM), either separately or in combination, are being increasingly used for genome-wide assessment of structural variants. Though there have been rapid improvements in these technologies over the past years, absence of a proper reference genome can hinder their utility to the fullest potential.

The GRCh38 reference genome, while widely used for variation identification, has many unresolved sequences and gaps, contributing to about 150 megabases of genome-wide ambiguity [4]. This encompasses regions in and around centromeres, telomeres, acrocentric p-arms, collapsed, and missing sequences. Consequently, GRCh38 can lead to numerous spurious variant calls, potentially hindering certain variant identification with accuracy [5]. In contrast, the T2T-CHM13 reference is haplotype-resolved, gapless, near-perfect, and offers substantial improvements in variant detection, particularly within these problematic regions [6].

OGM is a high-resolution imaging technique capable of detecting structural variants (SVs) >500 base pairs in length. This technology dubbed “Next-generation cytogenetics” is gaining traction in clinical laboratories for clinical SV identification in genetic diseases and cancers [7, 8]. However, adoption of T2T-CHM13 reference genomes for variant evaluation in OGM is still nascent in the clinical setup. To address this gap, our study aims to evaluate the utility of the T2T-CHM13 reference for OGM based SV detection in the context of genetic diseases.

## Clinical presentation and methodology

### Case1

A male child was diagnosed with severe Hemophilia due to factor VIII deficiency. A false negative PCR based assay for intron 22 inversion led to whole exome sequencing (WES) in the patient. WES could not identify any known pathogenic/ likely pathogenic variants in the F8 gene. To further investigate the cause of Hemophilia A in this patient, OGM was performed to identify any pathogenic structural variations.

### Case2

A 4-year-old male child, born to non-consanguineous parents, exhibited global developmental delay, facial dysmorphism and features of autism spectrum disorder. Karyotyping indicated an apparently balanced translocation between the long arm of chromosome 7 at cytoband 7q11.2 and chromosome 21 at cytoband 21q11.2. The parents had a history of three spontaneous first-trimester abortions. Trio OGM was done to identify the break point of the identified translocation and determine if there are any gene disruption or deletion or duplication events at that locus which can result in described phenotype and to look for any other pathogenic SV.

Ultra-high molecular weight genomic DNA was isolated from the patient’s blood using Bionano Prep SP Frozen Human Blood DNA Isolation Kit (Bionano, USA) followed by direct enzymatic labeling with fluorophores and imaging using Bionano Saphyr. The optical maps were then assembled and mapped to the two reference genomes GRCh38 and CHM13 T2T using the de novo assembly pipeline in Bionano Solve software version 3.8.0. The resultant structural variants were then assessed using Bionano Access version 1.8. A control SV dataset is provided by Bionano for both GRCh38 and CHM13 T2T reference genomes. This dataset encompasses structural variants from 394 individuals of varied ethnicities, and has been used for filtering out common variants. Comparisons of QC metrics and SV concordance between both the assemblies are provided in Tables S1 and S2, respectively.

## Results

### Case1

**Fig. 1:**
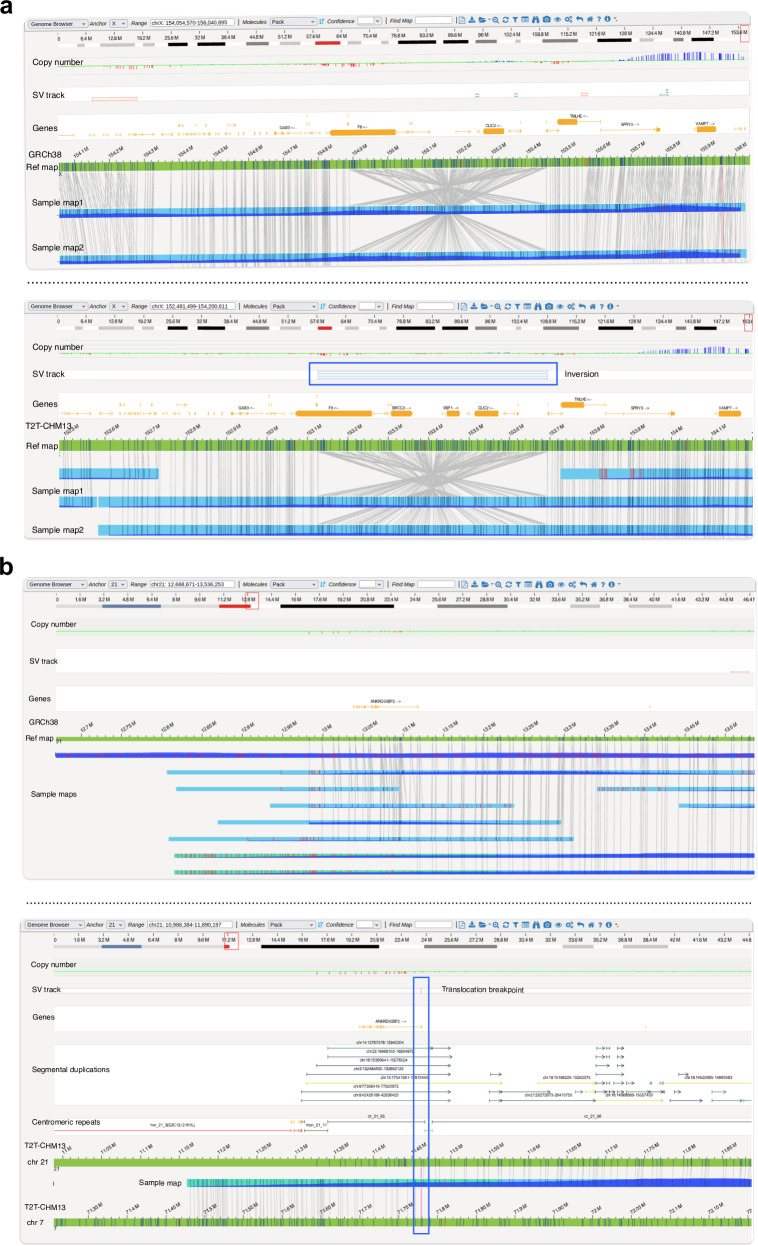
Improved SV detection in optical genome mapping using T2T-CHM13 reference. **a** Optical maps depicting the F8 Intron 22 inversion when mapped to GRCh38 (top) or T2T-CHM13 (bottom) references. Blue box highlights the SV call reported by Bionano Solve with the T2T-CHM13 reference. **b** Optical maps showcasing the pathogenic chr7-chr21 translocation. The variant call is highlighted with a blue box.

OGM of the proband using the GRCh38 reference genome identified 6181 structural variants via de novo genome assembly pipeline. Among these, 45 SVs were unique to the proband compared to the control database and encompassed insertions and deletions. Initial variant and visualization analysis did not reveal any structural variation in the F8 gene located on the X chromosome. However, manual inspection of consensus maps across the F8 gene region revealed the presence of a 0.56 Mb inversion, i.e., the intron 22 inversion (*Int22Inv*) (ogm[GRCh38] inv(X)(q28)(154888832_155456141)) which splits the F8 gene into two parts (Fig. 1a). The F8 inversion had a confidence score of 0.47, and hence was filtered out as it was below the pipeline’s confidence threshold of 0.7. Given the presence of the F8 gene in the subtelomeric region, which is challenging to resolve in GRCh38 [9], the T2T-CHM13 reference assembly was leveraged for a more accurate assessment of the inversion. This resulted in a significantly increased confidence score (0.82) for the *Int22Inv* variant. This enhanced score is attributable to the improved alignment of consensus maps to the left of the inversion breakpoint, as demonstrated by the RawConfidenceLeft score reported for T2T-CHM13 which had a twofold increase when compared to that of GRCh38 (Table 1). As the confidence score is calculated based on these raw confidence estimates, this suggests that mapping to the T2T-CHM13 reference genome leads to more reliable SV calls particularly in complex regions. The inversion was further confirmed using long distance PCR assay in-house (Fig. S1, Supplementary Methods).

**Table 1:** Confidence scores identified for clinically relevant structural variants.

Genome	Confidence	RawConfidenceLeft	RawConfidenceRight
**Intron 22 inversion (Int22Inv)**			
GRCh38	0.47	111.04	105.06
T2T-CHM13	0.82	**243.46**	111.8
**ogm[GRCh38] inv(X)(q28) (154888832_155456141)**			
GRCh38	0	23.35	15.94
T2T-CHM13	0.76	77.21	**6628.61**

Bold values show the significant changes in the raw confidence scores, which improved the variant detection.

### Case2

Consensus optical maps derived from the proband did not reveal the presence of a translocation aligned to the GRCh38 reference genome. We reviewed low confidence translocations below the recommended threshold of 0.02 which revealed presence of a translocation between chromosome 7 and 21 i.e.; t(7;21)(q11.22;q11.2) (ogm[GRCh38] t(7;21)(q11.22;q11.2)(70555391;13117484)) with a confidence score of 0, disrupting the AUTS2 gene. Given the association of AUTS2 in neurological disorders including autism spectrum disorder, intellectual disability and developmental delay, this finding was clinically significant [10, 11]. As the translocation breakpoint was in the pericentromeric region, consensus maps were realigned to the T2T-CHM13 reference genome. The translocation had a substantially increased confidence score of 0.76 due to improved alignment on either side of the breakpoint. This was evidenced by the RawConfidenceRight score of 6628.61 in T2T-CHM13 compared to 15.94 for the GRCh38 map (Table 1). Additionally, the RawConfidenceLeft score also showed a threefold increase with T2T-CHM13. Trio-optical genome mapping (Trio-OGM) analysis confirmed a de novo origin of the translocation in the proband. No other pathogenic SVs were identified. A closer look at the breakpoint location in chromosome 21 revealed an overlap with the centric transition region present in the pericentromeric region of the q arm in this acrocentric chromosome [12]. This region contained long tracts of segmental duplications which could cause ambiguous alignment (Fig. 1b). The same was confirmed by nanopore long read whole genome sequencing which showed the disruption of AUTS2 gene at chr7:70548861 (Fig. S2, Supplementary Methods). We hypothesize that multi mapping of molecules across highly similar segmental duplications in distinct genomic locations may lead to decreased confidence scores.

## Discussion

OGM has a significantly higher resolution than techniques such as karyotyping, fluorescence in situ hybridization and chromosomal microarray and can detect a wider range of SVs in a single assay. Its ability to identify cryptic inversions and translocations which cannot be detected by technologies mentioned above has a significant bearing on the recurrence risk of genetic anomalies in affected families. This study employed illustrative clinical cases to evaluate the robustness of the T2T-CHM13 reference genome in identifying clinically relevant variants within challenging genomic loci. We observed increased performance, particularly in repeat-rich regions like subtelomeric and pericentromeric loci containing segmental duplications, compared to existing reference genomes such as GRCh38. To our knowledge, this is the first optical genome mapping study demonstrating the enhanced performance of T2T-CHM13 for variant identification in these complex regions.

The widely used GRCh38 reference genome has more than 100 million unresolved and missing nucleotides across pericentromeric and subtelomeric regions, segmental duplications and ribosomal DNA (rDNA) arrays. In contrast, the T2T-CHM13 genome is a complete, gapless assembly and has an additional 200 million nucleotides which adds on to 8% of previously undiscovered sequence and corrects for many structural errors in complex regions. Multiple studies have reported limitations in detecting structural variants using the GRCh38 reference genome. These limitations stem from reference biases such as deletion bias due to assembly gaps, and inaccuracies in mapping complex regions, despite the availability of advanced SV detection technologies. Compared to GRCh38, the T2T-CHM13 reference genome has been shown to improve variant calling accuracy in medically relevant genes [6]. Furthermore, our study showcases that resolved repeat arrays and long stretches of segmental duplications within the T2T-CHM13 genome significantly improves alignment accuracy. This improvement in alignment, in turn, has the potential to enhance variant calling confidence scores. On the other hand, the lack of adequate control data and readily available annotation tracks in databases like DECIPHER, DGV (Database of Genomic variants), etc., which are important for clinical interpretation of structural variants are a hindrance to immediate adoption of T2T-CHM13 reference genome in clinical laboratories. Despite these limitations, we recommend the adoption of the T2T-CHM13 reference genome for resolving variants, particularly within challenging genomic regions.

## Supplementary information

Supplementary Material

## Data Availability

Data is available upon request.
