# Supplementary material for: Enhanced resolution of optical genome mapping utilizing telomere-to-telomere reference in genetic disorders
Source: Eur J Hum Genet. 2024 Dec 9;33(7):956–9. doi: 10.1038/s41431-024-01763-z (PMC12229659; doi:10.1038/s41431-024-01763-z)
Supplement: Supplementary file 1 — Supplementary Material [file 41431_2024_1763_MOESM1_ESM.docx]

**Supplementary Material**


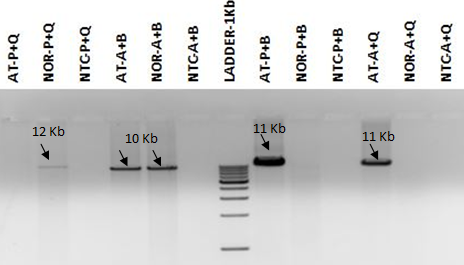


**Figure S1:** Long distance PCR to confirm the Factor VIII Intron 22 inversion identified by OGM. The methodology used for the PCR is as per Liu et al, 1998, and is described in detail below.


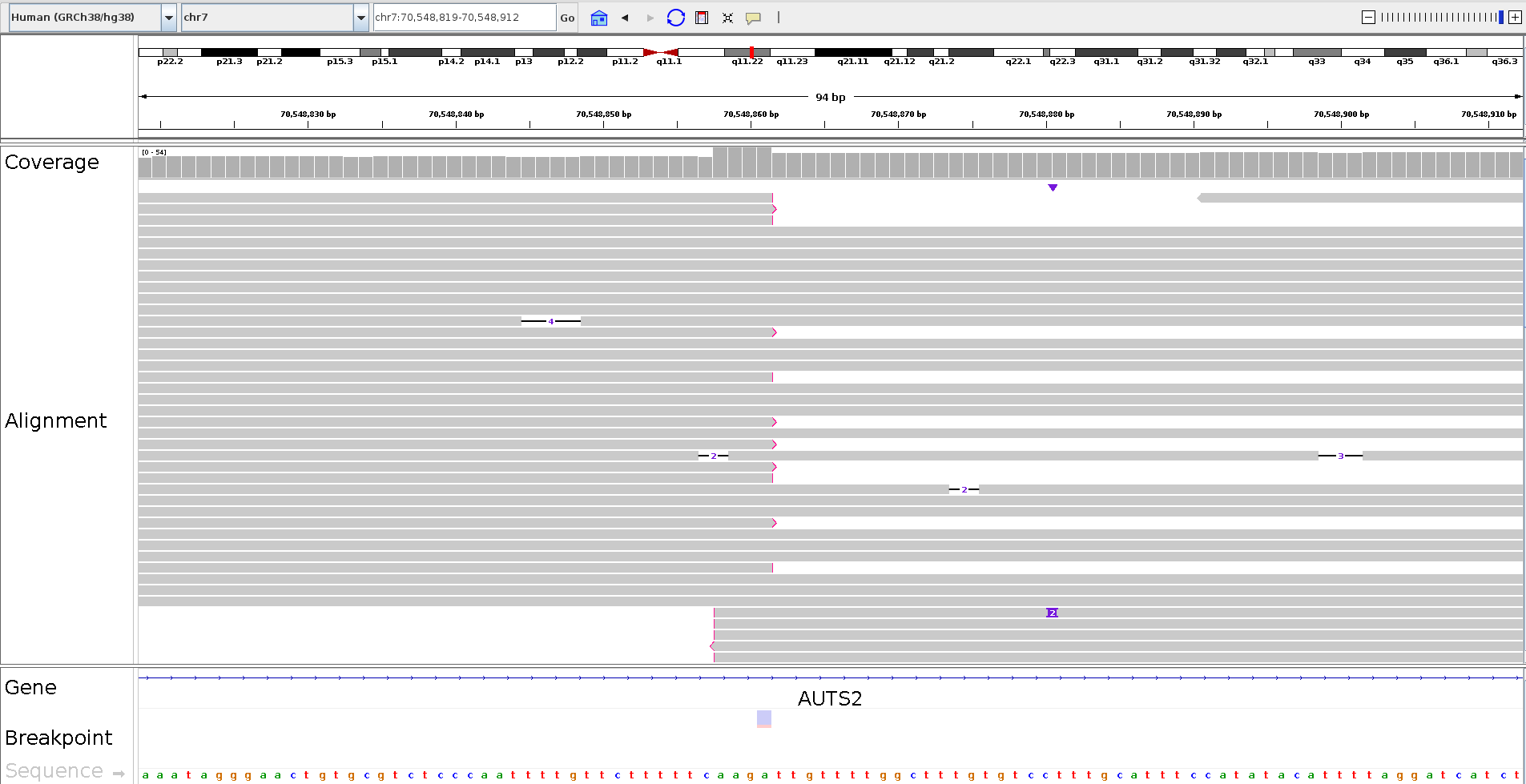


**Figure S2:** IGV screenshot showing translocation breakpoint in chr7:70548861 disrupting AUTS2 gene. Clipped ends are colored magenta to indicate SV breakpoints.

| **Metric** | **Case1** | | **Case2** | |
| --- | --- | --- | --- | --- |
| **Reference genome** | **GRCh38** | **T2T-CHM13** | **GRCh38** | **T2T-CHM13** |
| N50 (>= 150 kpb) | 227.25 | 227.25 | 216.32 | 216.32 |
| Map rate | 78.1 | 79.1 | 87 | 88 |
| Label density | 15.52 | 15.52 | 15.53 | 15.53 |
| Effective coverage | 367.31 | 368.55 | 405.96 | 406.81 |

**Table S1**: OGM QC metrics of cases 1 and 2

|  | **Case1** | | **Case2** | |
| --- | --- | --- | --- | --- |
| **Structural Variants** | **GRCh38** | **T2T-CHM13** | **GRCh38** | **T2T-CHM13** |
| **Insertion** | 4546 | 2184 | 4178 | 2136 |
| **Deletion** | 1742 | 1996 | 1805 | 1991 |
| **Inversion** | 112 | 65 | 90 | 42 |
| **Duplication** | 133 | 106 | 198 | 121 |
| **Inter-Translocation** | 2 | 79 | 1 | 90 |
| **Total** | 6535 | 4430 | 6272 | 4380 |

**Table S2**: Comparison of SVs called in GRCh38 and T2T-CHM13 assemblies

**Supplementary Methods**

**Validation of Factor VIII-Intron 22 Inversion detected on OGM by Long Distance PCR**

**Samples included in the assay[1]:**

| **Sl.No** | **Sample Id** | **DNA Concentration** |
| --- | --- | --- |
| 1 | Case 1 (AT) | 33 ng/µL |
| 2 | Normal/ Negative Control (NOR) | 152 ng/µL |
| 3 | No Template Control (NTC) | - |

**Primers:**

| **Sl.No** | **Primer Id** | **Sequence (5’-3’)** | **Target in the genomic DNA** |
| --- | --- | --- | --- |
| 1 | P | GCCCTGCCTGTCCATTACACTGATGACATTATGCTGAC | Intron 22 of the Factor VIII gene (Int22h1) |
| 2 | Q | GGCCCTACAACCATTCTGCCTTTCACTTTCAGTGCAATA |  |
| 3 | A | CACAAGGGGGAAGAGTGTGAGGGTGTGGGATAAGAA | Homologues of the intron 22 of the Factor VIII gene at the Xq telomere (Int22h2, Int22h3 ) |
| 4 | B | CCCCAAACTATAACCAGCACCTTGAACTTCCCCTCTCATA |  |

**Reagent Preparation for PCR:**

| **Sl.No** | **Reagent** | **1X** | **3X** |
| --- | --- | --- | --- |
| 1 | TaKaRa-Prime STAR GXL Premix (2X) | 12.5 µL | 37.5 µL |
| 2 | Di-Methyl Sulfoxide | 2 µL | 6 µL |
| 3 | Forward Primer | 1.5 µL | 4.5 µL |
| 4 | Reverse Primer | 1.5 µL | 4.5 µL |
| 5 | Template DNA | ~ 100 ng/reaction | |
| 6 | Nuclease Free Water | Make volume upto 25 µL/reaction | |

**Primer Combinations Used: P+Q, A+B (for wild type); P+B, A+Q (for inverted segments)**

**Thermal Profile:**

| **Step** | **Temperature** | **Time** | **No of cycles** |
| --- | --- | --- | --- |
| Denaturation | 98°C | 10 seconds | 30 |
| Annealing  +  Extension | 68°C | 10 minutes |  |

**Agarose Gel Electrophoresis:**

10µL of the amplicon was loaded onto a 0.8% agarose gel stained with Ethidium bromide.

**Bands expected on an agarose gel:**

| **Primers** | **Normal** | **Carrier** | **Affected** |
| --- | --- | --- | --- |
| **P+Q** | **12 Kb** | **12 Kb** | **None** |
| **A+B** | **10 Kb** | **10 Kb** | **10 Kb** |
| **P+B** | **None** | **11 Kb** | **11 Kb** |
| **A+Q** | **None** | **11 Kb** | **11 Kb** |

**Validation of AUTS2 gene disruption using whole genome long read sequencing**

The high molecular weight DNA isolated for OGM in case 2 was also used for long read sequencing. Nanopore libraries were prepared using Ligation Sequencing Kit V14 (Oxford Nanopore, UK) according to the manufacturer’s protocol. In brief, the genomic DNA was treated with NEBNext Ultra II End Repair/dA-Tailing Module and NEBNext FFPE DNA Repair Mix (NEB, US). The samples were purified using Ampure XP beads (Beckman Coulter, US) and nanopore sequencing adapters were ligated to the DNA fragments. Samples were purified once more and the library was quantified using Qubit (ThermoFisher Scientific, US). 50 fmol of library was loaded onto a R10.4.1 PromethION flow cell and sequenced for 72 hrs.

The raw data in POD5 format was basecalled using dorado v0.8.0 in super-accuracy mode (2). The data quality was assessed using NanoPlot (3). Reads in FASTQ format were aligned to the GRCh38 reference genome using winnowmap2 (4). The aligned bam file was sorted and indexed using samtools (5), and structural variant identification was done using Sniffles2 (6). The bam file was also loaded onto the IGV browser (7) for manual inspection of the AUTS2 breakpoint.

**References**

1. Liu Q, Sommer SS. Subcycling-PCR for multiplex long-distance amplification of regions with high and low GC content: application to the inversion hotspot in the factor VIII gene. Biotechniques. 1998;25:1022-8. <https://doi.org/10.2144/98256rr01>

2. Oxford Nanopore Technologies. Dorado: a high-performance, easy-to-use, open source basecaller for Oxford Nanopore reads. 2024. <https://github.com/nanoporetech/dorado/tree/release-v0.8>

3. De Coster W, Rademakers R. NanoPack2: population-scale evaluation of long-read sequencing data. Bioinformatics 39: btad311.

4. Jain C, Rhie A, Hansen NF, Koren S, Phillippy AM. Long-read mapping to repetitive reference sequences using Winnowmap2. Nature Methods. 2022 Jun;19(6):705-10.

5. Danecek P, Bonfield JK, Liddle J, Marshall J, Ohan V, Pollard MO, *et al.* Twelve years of SAMtools and BCFtools. Gigascience. 2021 Feb;10(2):giab008.

6. Smolka M, Paulin LF, Grochowski CM, Horner DW, Mahmoud M, Behera S, *et al.*  Detection of mosaic and population-level structural variants with Sniffles2. Nature biotechnology. 2024 Jan 2:1-0.

7. Robinson JT, Thorvaldsdóttir H, Winckler W, Guttman M, Lander ES, , *et al.* Integrative genomics viewer. Nature biotechnology. 2011 Jan;29(1):24-6.
